# A case report: white cord syndrome following anterior cervical discectomy and fusion: importance of prompt diagnosis and treatment

**DOI:** 10.1186/s12891-020-3162-3

**Published:** 2020-03-12

**Authors:** Deuk Soo Jun, Jong-Min Baik, Seung-Kwan Lee

**Affiliations:** Department of Orthopedic Surgery, Gil Medical Center, Gachon University College of Medicine, University of Gachon, 21, Namdong-daero 774, Namdong-gu, Incheon, Republic of Korea

**Keywords:** Cervical, Huge disc, discectomy, Fusion, White cord syndrome

## Abstract

**Background:**

Objective: White cord syndrome is extremely rare and search of the literature has revealed very few cases. Postoperative MR scan revealed hyperintense intrinsic cord signal changes within cord ischemia and edema. It is thought to be caused by reperfusion injury of the spinal cord. This is called white cord syndrome. This report is very rare case of ‘White Cord Syndrome’ with paraplegia after anterior cervical discectomy and fusion (ACDF).

**Case presentation:**

A 49-year-old woman presented with neck pain lasting for several months and second and third finger radiating pain. Severe cervical herniated intervertebral disc findings could be identified at C6–7 level on C-spine MRI. ACDF C6–7 surgery was performed. Immediately after the operation, physical examination revealed paraplegia and emergency MRI was performed. On MR images, T2 high signal myelopathy suspected as reperfusion injury at C6–7 level, and emergency surgery was performed under diagnosis of white cord syndrome. After the emergency operation, the paraplegic problem was gradually resolved. Before discharge, motor power and sensory deficit of bilateral lower extremity were fully recovered.

**Conclusion:**

Surgeons should explain the possibility of white cord syndrome before cervical decompression surgery and should perform a neurological examination immediately after surgery. We recommend that the importance of early recognition and prompt treatment of white cord syndrome.

## Background

Anterior cervical discectomy and fusion (ACDF) is a common surgical procedure for cervical spine surgery and has resulted in good clinical results. The most serious complication after cervical spine surgery is neurologic deficit such as paralysis or paraplegia. This paraplegia is reported to be mostly caused by hematoma or iatrogenic injury. However, there is paraplegia that cannot be explained by these things, which can be caused by reperfusion injury, so called ‘white cord syndrome’ [1]. White cord syndrome is extremely rare and search of the literature has revealed very few cases. Postoperative MR scan revealed hyperintense intrinsic cord signal changes within cord ischemia and edema. It is thought to be caused by reperfusion injury of the spinal cord. Although the mechanism of the disease is not clear, it is believed that free radical oxygen damages the spinal cord [2–4]. We report a case of this complication after ACDF.

## Case presentation

**Fig. 1:**
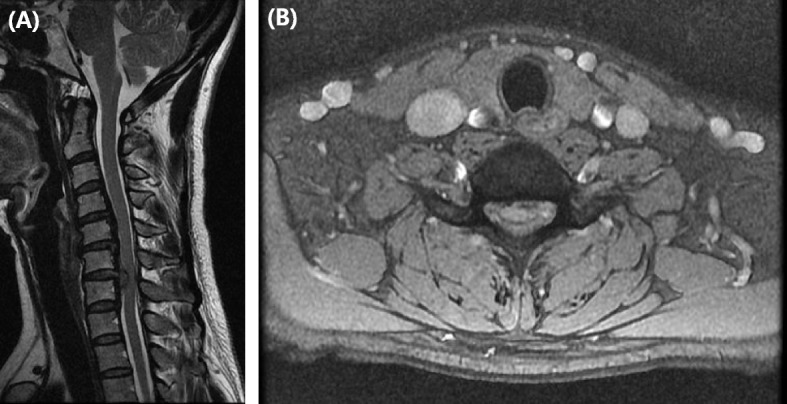
**a** Preoperative sagittal T2 weighted MR images showing large central disc extrusion with inferior migration, severe cord compression on C6–7 level. **b** Preoperative axial T2-weighted MRI showing severe C6–7 cord compression by a massive disc herniation

**Fig. 2:**
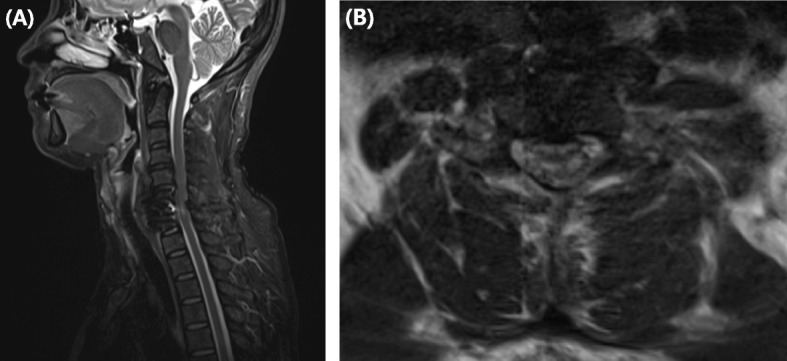
**a** Immediate postoperative sagittal STIR (Short tau inversion recovery) MR image shows cervical anterior decompression state and high signal intensity at C6–7 level. **b** Immediate postoperative axial T2 weighted MR image shows anterior bilateral symmetric ovoid foci of high signal intensity at C6–7 level

**Fig. 3:**
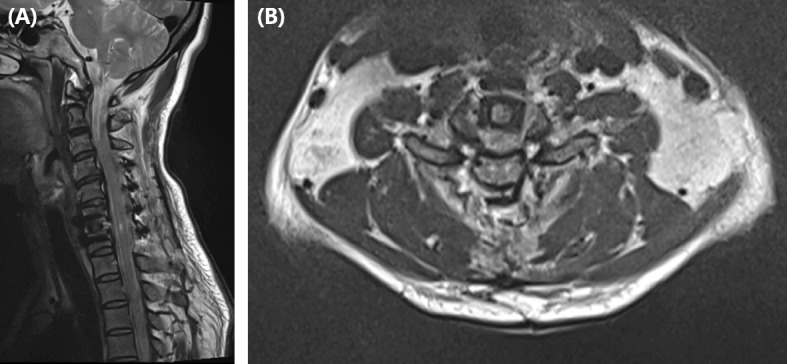
**a** One week postoperative sagittal T2 weighted MR image showing high intensity patchy signal changes within the cord with cord swelling as called White cord syndrome. **b** One week postoperative axial T2 weighted MR Image showing 2 points high signal intensity at C6–7 level

This 49-year-old woman presented with neck pain lasting for several months and second and third finger radiating pain. She had no neurological deficit except for severe radiating pain of C6 dermatome. And the myelopathic neurologic examinations were uneventful, such as Hoffman’s sign, knee jerk, and ankle clonus. There was no past history other than hypertension and no relevant past medical interventions. On the C-spine MRI, we were able to confirm that the cord was compressed by extrusion of the large central disc at the C6–7 level (Fig. 1a, b). ACDF C6–7 surgery was performed and the cord was decompressed by removing the C6–7 disc material. The interbody cage was inserted into the C6–7 disc space and the plate (Vectra™, Depuy Synthes, CH) was fixed on the C6–7 body anterior surface. On immediately postoperative physical examination, the upper extremity motor and sensory nerves were normal however, ankle clonus 4+, knee jerk 4+, sensory deterioration, and bilateral lower extremity motor grade 0 were confirmed. C-spine MRI was taken immediately. Wide spreading high signal intensity in sagittal STIR (Short tau inversion recovery) MR image was observed at C6–7 level (Fig. 2a). In axial T2-weghted MR, anterior bilateral symmetric ovoid foci of high signal intensity (the snake-eyes sign) was observed (Fig. 2b). She was started high dose steroid (Methylprednisolone 30 mg/kg/15 min + 5.4 mg/kg/23 h) was administered under white cord syndrome diagnosis and emergency operation was performed. She underwent laminoplasty (Centerpiece™, Medtronic, USA) operation at C4–5–6-7 levels. The T2 weighted MR scan was repeated 1 week later as seen in Fig. 3a, b. After surgery, motor and sensory deficits were gradually resolved. Before discharge, motor power and sensory deficit of bilateral lower extremity were fully recovered. However, mild tingling sensation was remained at bilateral L5, S1 dermatome area. The patient had been discharged at 14 days postoperatively, and the preoperative symptoms almost disappeared (Table 1).

**Table 1:** Neurological assessment: American Spinal Injury (ASIA) scale

		Motor Gr (Rt/Lt)	Sensory Gr (Rt/Lt)	ASIA impairment scale
Before first operation	L2, Hip flexors	5/5	2/2	E
	L3, Knee extensors	5/5	2/2	
	L4, Ankle dorsiflexors	5/5	2/2	
	L5, Long toe extensiors	5/5	2/2	
	S1, Ankle Plantar flexors	5/5	2/2	
After first operation	L2, Hip flexors	0/0	0/0	A
	L3, Knee extensors	0/0	0/0	
	L4, Ankle dorsiflexors	0/0	0/0	
	L5, Long toe extensiors	0/0	0/0	
	S1, Ankle Plantar flexors	0/0	0/0	
After second operation (1 day)	L2, Hip flexors	4/4	1/1	D
	L3, Knee extensors	4/4	1/1	
	L4, Ankle dorsiflexors	4/4	1/1	
	L5, Long toe extensiors	4/4	1/1	
	S1, Ankle Plantar flexors	4/4	1/1	
After second operation (14 days)	L2, Hip flexors	5/5	2/2	E
	L3, Knee extensors	5/5	2/2	
	L4, Ankle dorsiflexors	5/5	2/2	
	L5, Long toe extensiors	5/5	2/2	
	S1, Ankle Plantar flexors	5/5	2/2	

## Discussion and conclusions

White cord syndrome is extremely rare and search of the literature has revealed very few cases. Chin at al [5]. reported the case of a 59-year-old man affected by C5–6 severe cord compression, who underwent C4–5 and C5–6 ACDF surgery. Tetraplagia occurred in the patient after surgery, and he was treated with more extensive decompression and steroids immediately. The patient was partially improved. And Giammalva et al. [6] reported the case of a 64-year-old man affected by C4–6 severe cord compression, who underwent C3–4 and C5–6 ACDF. Tetraparesis occurred in the patient after surgery, and the patient was immediately started only high-dose steroid protocol (NASCIS III). The patient was partially improved. Antwi et al. [7] reported the case of a 68-year-old man affected by C4–6 severe cervical stenosis, who underwent C3–7 posterior decompression surgery. This was the first white cord syndrome case caused by posterior cervical operation. Hemiparesis occurred in the patient after surgery, and the patient was immediately started high-dose steroid protocol. But, the patient was partially improved. The precise mechanism of development of the white cord syndrome has not been reported. However, it is presumed that reperfusion injury occurs in chronically ischemic tissue caused by free radical oxygen. Hall E [8]. reports that the use of high-dose steroids inhibits the attack of free radical oxygen.

In our case, the patient had chronic severe cord compression, and the result of cord decompression was an unexpected neurological deficits. Postoperative T2-weighted MRI showed intramedullary hyperintensity and cord swelling. So we used high-dose steroids to reduce oxidative damage and performed extensive decompression to relieve localized cord expansion. However, there is insufficient evidence to suggest that this has helped the patient improve symptoms. So we performed immediately surgery as laminoplasty at C4–5–6-7. The reason for choosing laminoplasty was to perform a quick operation and to decompress more widely while saving the neck motion (Table 2). We were convinced that there was no injury during surgical procedure, and we could conclude that neurological deficit occurred as an unknown cause, that is, reperfusion mechanisms. This is mentioned in some literature and is due to oxidative stress, although the exact cause is not known.

**Table 2:** Demographics and Clinical Characteristics of other cases

Case	Age/Sex	Diagnosis	Treatment	Symptom after first operation	Treatment after first operation	Final outcome
Chin et al.	59 / Male	CSM	ACDF C4–5, C5–6	Incomplete tetraplegia	1. High-dose steroid protocol 2. C5 corpectomy	Left finger flexion 3/5 Left finger extension and interossei 4/5 Left hip abduction 5−/5 Left quadriceps and hamstring 4/5 Left other muscle groups 2/5 Right lower limb 5/5
Giammalva et al.	64 / Male	CSM	ACDF C3–4, C5–6	Tetraparesis	1.High dose steroid protocol	Right hand prehension 3/5 Right arm flexion 2/5 Both leg flexion 2/5
Antwi et al.	68 / Male	CSM	PCDF C3–7	Hemiparesis (Left)	1. High-dose steroid protocol	Left wrist flexion 3/5 Left wrist extension 4+/5 Left elbow flexion 4+/5 Left elbow extension 4+/5 Left hip flexion 2+/5 Left knee extesion 4/5 Left ankle dorsiflexion 1/5 Left ankle plantar flexion 2/5
Our case	49 / Female	CSR	ACDF C6–7	Paraplegia	1. High-dose steroid protocol 2. Laminoplasty C4–5–6-7	Both lower limb full strength

^a^*CSM* Cervical spondylotic myelopathy, *CSR* Cervical spondylotic radiculopathy, *ACDF* Anterior cervical discectomy and fusion, *PCDF* Posterior cervical decompression and fusion

In conclusion, High-dose steroids and extensive decompression surgery in post-operative white cord syndrome is controversial but we think it is one of the available treatments. And also surgeons should adequately explain and give warning the possibility of complication, such as white cord syndrome before surgery and check the neurological examination immediately after surgery in patient with severe cord compression. We recommend that the importance of early recognition and prompt treatment of white cord syndrome.

## Data Availability

The datasets used and/or analyzed during the present study are available from the corresponding author on reasonable request.

## Abbreviation

ACDF: Anterior cervical discectomy and fusion
